# GABA Regulates the Multidirectional Tangential Migration of GABAergic Interneurons in Living Neonatal Mice

**DOI:** 10.1371/journal.pone.0027048

**Published:** 2011-12-13

**Authors:** Hiroyuki Inada, Miho Watanabe, Taku Uchida, Hitoshi Ishibashi, Hiroaki Wake, Tomomi Nemoto, Yuchio Yanagawa, Atsuo Fukuda, Junichi Nabekura

**Affiliations:** 1 Department of Developmental Physiology, National Institute for Physiological Sciences, Okazaki, Japan; 2 Department of Physiological Sciences, The Graduate University for Advanced Studies, Okazaki, Japan; 3 Department of Physiology, Hamamatsu University School of Medicine, Hamamatsu, Japan; 4 Laboratory of Molecular and Cellular Biophysics, Research Institute for Electric Science, Hokkaido University, Sapporo, Japan; 5 Department of Genetic and Behavioral Neuroscience, Graduate School of Medicine, Gunma University, Maebashi, Japan; Institut National de la Santé et de la Recherche Médicale, France

## Abstract

Cortical GABAergic interneurons originate from ganglionic eminences and tangentially migrate into the cortical plate at early developmental stages. To elucidate the characteristics of this migration of GABAergic interneurons in living animals, we established an experimental design specialized for *in vivo* time-lapse imaging of the neocortex of neonate mice with two-photon laser-scanning microscopy. In vesicular GABA/glycine transporter (VGAT)-Venus transgenic mice from birth (P0) through P3, we observed multidirectional tangential migration of genetically-defined GABAergic interneurons in the neocortical marginal zone. The properties of this migration, such as the motility rate (distance/hr), the direction moved, and the proportion of migrating neurons to stationary neurons, did not change through P0 to P3, although the density of GABAergic neurons at the marginal zone decreased with age. Thus, the characteristics of the tangential motility of individual GABAergic neurons remained constant in development. Pharmacological block of GABA_A_ receptors and of the Na^+^-K^+^-Cl^−^ cotransporters, and chelating intracellular Ca^2+^, all significantly reduced the motility rate *in vivo*. The motility rate and GABA content within the cortex of neonatal VGAT-Venus transgenic mice were significantly greater than those of GAD67-GFP knock-in mice, suggesting that extracellular GABA concentration could facilitate the multidirectional tangential migration. Indeed, diazepam applied to GAD67-GFP mice increased the motility rate substantially. In an *in vitro* neocortical slice preparation, we confirmed that GABA induced a NKCC sensitive depolarization of GABAergic interneurons in VGAT-Venus mice at P0-P3. Thus, activation of GABA_A_R by ambient GABA depolarizes GABAergic interneurons, leading to an acceleration of their multidirectional motility *in vivo*.

## Introduction

The establishment of CNS cytoarchitectures is essential for brain function. To acquire ordered neuronal arrangements, neurons migrate from their birth place to their final positions during development. The function of the cerebral cortex essentially relies on the balance between excitation and inhibition, which is served by two classes of neurons: excitatory glutamatergic projection neurons and inhibitory GABAergic interneurons. Glutamatergic neurons arise in the germinal zones lining the lateral ventricle, and migrate radially within the cortex toward the pial surface [1]. On the other hand, most cortical GABAergic interneurons originate from the ganglionic eminences (GEs), located in the subpallial telencephalon [2]. These GABAergic interneurons migrate tangentially toward the pallium and cortex. They show several migration modalities in the neocortex before they reach their final positions in their corresponding target layers [3]; 1) tangential migration along the intermediate zone (IZ), 2) radial migration from the IZ towards the marginal zone (MZ) via the cortical plate (CP) [4] or from the IZ towards the ventricle surface [5], 3) multidirectional tangential migration in the MZ [6], and 4) radial migration from the MZ toward the CP [7]. The proper regulation of each of these modalities has a critical role in determining the final location of GABAergic interneurons in the neocortex [8]. In particular, disruption of intracortical migration in the MZ causes changes in the distribution of interneurons in the mature cortex [9], [10]. This suggests that multidirectional tangential migration in the MZ has a critical role for the balanced distribution of inhibitory neurons throughout the entire cortical areas. It has been reported that the tangential migration of GABAergic interneurons mainly proceeds during embryonic period [11]. In consistent with this finding, the interneurons in the IZ disappear by P0.5 [12]. On the other hand, it has been reported that a significant number of interneurons still exist in the MZ at P0.5 [13]. These reports imply that the MZ is one of the dominant cortical layers for the migration of GABAergic interneurons during early postnatal development. Thus, *in vivo* examination of migratory behavior and its regulatory mechanism in the early postnatal MZ is important to understand the organizational principles of the neocortex.

Extracellular GABA has been reported to have two contrary roles in regulating the migration of cortical GABAergic interneurons. On the one hand, time-lapse imaging in an *in vitro* embryonic preparation revealed that extracellular ambient GABA promoted tangential migration from the medial GEs towards the neocortex via the tonic activation of GABA_A_Rs [14]. Alternatively, *in vivo* local application of GABA_A_R-agonists and antagonists via Elvax implants induced severe disturbances in cortical layering and focal cortical dysplasias, suggested the modulation of GABA_A_R-mediated mechanisms impaired radial migration of cortical GABAergic interneurons [15]. An intrinsic change in the responsiveness of migrating neurons to GABA, from acceleration migration to inhibiting migration, mediated via the upregulation of the potassium-chloride cotransporter KCC2 was recently reported using an embryonic slice culture system [16]. Taken together, this suggests that extracellular GABA may promote tangential migration and terminates radial migration in the cortex. However, it is not known exactly whether GABA promotes or inhibits the multidirectional tangential migration in the MZ *in vivo*, which determines the final distribution of GABAergic neurons across the cortical areas. Furthermore, the underlying GABA action (excitation or inhibition) mediating any affects *in vivo*, which can depend on extracellular GABA [17] and intracellular Cl^−^ concentration [18], is also unclear. It is necessary to elucidate the GABA action on the migrating neurons in intact preparation and thus, in the present study, we carried out *in vivo* time-lapse imaging of GABAergic interneurons in the MZ using two-photon microscopy. We found *in vivo* that interneurons migrate in all directions within the tangential plane, and that the activation of GABA_A_Rs enhances this motility, by increasing the frequency of movements, without affecting the preference in direction and stroke of each movement.

## Results

### Development of *in vivo* imaging for immature brain

**Figure 1:**
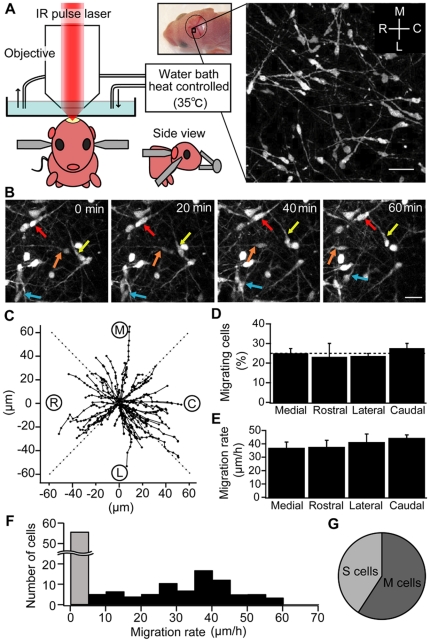
Multidirectional tangential migration of GABAergic interneurons in the MZ. (A) Left: Schematic drawing of the *in vivo* imaging setup optimized for immature mouse. A mouse is restrained under the microscope using three steel bars attached on the back, right and left sides of the head. The temperature of the immersion water is kept at 35°C. The panel shows a stereomicroscopic image from a P0 mouse indicates the imaging area for the migration studies. Right: Two-photon image of Venus positive cells in the MZ *in vivo*. Scale bar = 20 µm. (B) Time-lapse images of migrating neurons in the MZ. Each image is the sum of 10×0.5 µm sections advancing in the z axis (total thickness 5 µm). Times shown at the upper right represent the time elapsed after starting the observation. Four single GABAergic interneurons are marked by red, orange, yellow, and blue arrows. Scale bar = 15 µm. (C) The trajectory of migrating neurons in VGAT-Venus (n = 61 cells, 5 animals at P0–P3). Each black circle represents the location of each neuron defined as the basal portion of the leading process. The start position of each neuron is normalized to the coordinate center. The time interval between each black circle is 3 minutes, and the total imaging time is 1 hour. Labels and dotted lines indicate the 4 direction sectors: M: medial; C: caudal; L: lateral; R: rostral. (D) The proportion of neurons migrating in each direction. The dotted line is drawn at the 25% level. If the population of neurons migrates without any preference in direction, the proportion of neurons migrating in each direction should be close to 25%. Each column indicates mean ± SEM. (E) Cell displacement during 1 hour in each direction. Each column indicates mean ± SEM. (F) Histogram of the migration rate. Gray bar indicates the number of stationary cells, which migrate less than 5 µm during 1 hour. (G) A pie chart indicating the mean proportion of cells showing moving or stationary behavior.

*In vivo* imaging of neuronal population with single-cell resolution became possible with the application of two-photon laser-scanning microscopy [19], [20]. While the importance of *in vivo* experiments in immature animals to understand the development of neural circuit is well accepted, there are also studies which examined the dynamics of neural circuits during development [21], [22]. Our initial studies found that the standard approaches to make a cranial window (open skull or thin skull methods) needed for imaging, easily damaged the migrating interneurons in the MZ. Furthermore, the application of room temperature immersion water at the skull – lens interface disturbed the local circulation at the brain surface. Thus, we performed two technical improvements for imaging cortical neurons of immature mice (Fig. 1A). First, we circulated warm water (35°C) above the imaging area to prevent any decrease of cortical temperature. Secondly we made a three-direction restraint bar (back, left, and right side of the head) to alleviate brain movement while avoiding any damage due to pressure. At the same time, the thin skull bone of immature mouse allowed the sufficient transcranial access of the laser without the need for any surgery. With these combined technical improvements, we succeeded in the stable time-lapse imaging of the migration of cortical GABAergic interneurons located in the MZ of immature living mice.

### Multidirectional tangential migration of GABAergic interneurons in immature intact cortex

Migratory behavior of GABAergic interneurons in the MZ has been reported using a flat-mount cortical preparation *in vitro* [6] and *in vivo* [7]. In the present study, we observed GABAergic interneurons in the MZ *in vivo* by applying two-photon time-lapse imaging to VGAT-Venus transgenic mice (VGAT-Venus mice) at P0–P3. In the neocortex of VGAT-Venus mice, more than 95% of Venus positive cells expressed GABA and more than 93% of GABA-immunoreactive cells were positive for Venus [23]. Thus it is unlikely that only a specific subtype of GABAergic interneurons were observed in the present experiment.

We performed continuous time-lapse imaging (1 frame/3 min, Fig. 1B) for at least 60 minutes and made a quantitative analysis of the migration direction. By plotting the translocation of a basal point of the leading processes in each frame, we quantified the movement of each GABAergic interneuron (Fig. 1C, n = 61 cells, 5 animals). Neurons that migrated out of the focal plane during the imaging period (less than 10% of neurons) were excluded from subsequent analysis. Migrating neurons were defined as those that migrated more than 5 µm during 1 hour. We subdivided these migrating neurons into 4 groups according to their final position, and counted the proportion of the migrating neurons located in each sector (Fig. 1D). Neurons migrated equally in each direction (medial; 24.9±2.5%, rostral; 23.1±7.0%, lateral; 23.5±1.4%, caudal; 27.5±2.6%). Furthermore, the migrating rate was similar among the 4 directions (Fig. 1E, medial 36.8±4.5 µm, rostral 37.5±5.2 µm, lateral 41.1±6.3 µm, caudal 44.2±2.5 µm, for 1 hour. p>0.05). These results indicate that GABAergic interneurons in the MZ have no preference in direction of migration within the tangential plane *in vivo*. The histogram of migration rate showed a broad distribution among all the neurons (Fig. 1F), and nearly half of them did not migrate significantly (Fig. 1G, 59.2±9.5% for moving cells and 40.8±3.0% for stationary cells).

When we compared the migration rate between P0 and P3 VGAT-Venus mice including control and ACSF treated groups, there was no significant difference between two groups (30.9±1.5 µm/hr for P0 and 36.9 µm/hr for P3 VGAT-Venus mice, n = 3 animals respectively). The cell density in the imaging plane decreased between P0 and P3 in VGAT-Venus mice (1063±201 cells/mm^2^ in P0 and 696±46 cells/mm^2^ in P3 MZ) without any change in the proportion of stationary cells (35.4±2.6% for P0 and 37.6±2.5% for P3 mice).

### Involvements of GABA_A_R and [Cl^−^]i in multidirectional migration

**Figure 2:**
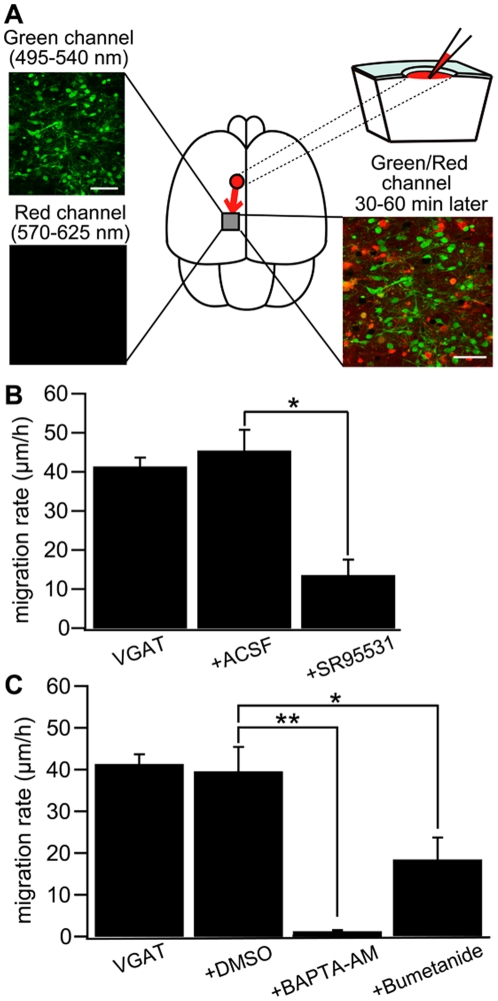
*In vivo* drug application in immature cortex. (A) Upper left: Two-photon image of Venus positive cells immediately after drug application in a P0 VGAT-Venus mouse. Making a hole at 1–2 mm away from the observed area has no effect on the morphology of the cells within imaging area. Scale bar = 50 µm. Lower left: Two-photon image of sulforhodamine 101 (SR101) showing that the drug does not reach the imaging area immediately after injection. Upper right: Schematic drawing of the drug injection procedure; making a hole and injecting the drug conjugated with SR101. The drug diffuses into the imaging area after 30–60 minutes of injection. Lower right: Two-photon image of Venus-positive cells and SR101 showing that the pipette solution spread around the cells without injuring them. Scale bar = 50 µm (B) Cell displacement during 1 hour of migration for neurons in VGAT-Venus without drug application (VGAT), after injection of ACSF (+ ACSF) and after injection of SR95531 (+SR95531). The selective GABA_A_R antagonist (SR95531, 1 mM, 1 µl) reduced the migration rate significantly (*p<0.05) whereas ACSF (1 µl) had no effect. (C) DMSO (1 µl) had no effect on cell displacement. Chelating intracellular Ca^2+^ by BAPTA-AM (2 mM, 1 µl) conjugated with DMSO almost completely inhibited the migration (**p<0.01) and blockade of NKCC by bumetanide (1 mM, 1 µl) significantly reduced the migration rate (*p<0.05). VGAT-Venus; n = 5 animals, ACSF; n = 5 animals, DMSO; n = 4 animals, SR95531; n = 5 animals, bumetanide; n = 4 animals, BAPTA-AM; n = 4 animals. All animals were used at P0–P3, except the bumetanide group which were used at P1–P3. Each column in B and C indicates mean ± SEM.

To investigate the regulatory mechanisms of these migratory behaviors of GABAergic interneurons, we developed *in vivo* drug application system for immature cortex (Fig. 2A). Drugs were pressure-injected approximately 2 millimeters away from the imaging area via a glass pipette inserted through a small hole in the skull. This procedure did not induce any noticeable damage to neurons in the imaging area, and preliminary data (not shown) indicted no increase in cleaved caspase-3 immunostaining following saline pressure-injection. To examine whether the drugs applied could be delivered to imaging areas, sulforhodamine 101 (SR101), a red fluorescent molecule that has been used to label cortical glial cells [24], was also added to the solution. We confirmed that drugs could access the imaging area by detecting this SR101 fluorescence after similar pressure injection. Application of artificial cerebrospinal fluid (ACSF) had no effect on the migration rate of cortical GABAergic interneurons (Fig. 2B, control 41.3±2.4 µm/hr, ACSF 45.3±5.4 µm/hr. p>0.05). To investigate the role of GABA signaling in this multidirectional migration, we applied SR95531, a selective GABA_A_R antagonist. Injection of SR95531 (1 mM, approximately 1 µl) reduced the migration rate significantly compared with that of ACSF injected group (Fig. 2B, SR95531 13.5±4.1 µm/hr. p<0.05). This finding suggests that endogenous GABA_A_R agonists, e.g. GABA, continuously activate GABA_A_R to promote increased migration rate. In addition, chelating [Ca^2+^]_i_ by injecting BAPTA-AM (dissolved in DMSO) virtually abolished migration rate compared with DMSO injection group (Fig. 2C, DMSO 39.5±5.9 µm/hr, BAPTA-AM 1.2±0.3 µm/hr. p<0.01). Furthermore, bumetanide, a blocker of the Na^+^-K^+^-Cl^−^ cotransporters (NKCC), also reduced migration rate significantly (Fig. 2C, DMSO alone; 39.5±5.9 µm/hr, bumetanide; 18.2±5.2 µm/hr). Taken together, the data suggest that the activity of GABA_A_R, [Ca^2+^]_i_ and [Cl^−^]_i_ levels regulate the motility of multidirectional tangential migration, either separately or in an associated manner. A previous study reported that the activation of GABA_A_R could depolarize the membrane potential of immature neurons due to the activity of NKCC1 [25].

### Gramicidin patch-clamp recording from GABAergic interneurons in slice preparations

**Figure 3:**
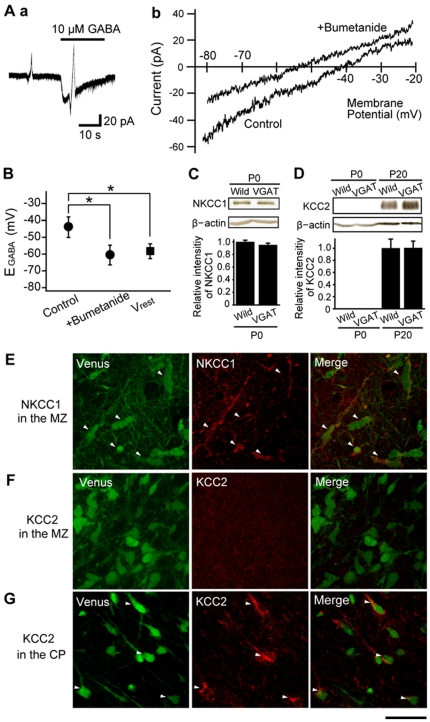
Depolarizing effect of GABA caused by expression of NKCC1. (A) Left panel: Current responses to ramp voltage steps in the absence and presence of 10 µM GABA. Voltage-clamp recordings were performed at a holding potential (V_H_) of −60 mV, with ramp voltage step pulses from −80 mV to −20 mV with a 3 second duration. Gramicidin perforated patch recordings were done on Venus positive cells using a slice preparation from P0–P3 VGAT Venus mice. Right panel: Samples of GABA_A_R current-voltage (I–V) curves in control conditions and in the presence of 10 µM bumetanide. E_GABA_ was estimated by the intersection of the voltage axis of each curve. (B) Mean E_GABA_ in control conditions and in the presence of bumetinide. The mean resting membrane potential (V_rest_), obtained in current clamp mode, is also shown. The more negative value of V_rest_ as opposed to E_GABA_ in control conditions indicates a depolarizing effect of GABA (*p<0.05). Bumetanide, a NKCC1 blocker significantly shifted E_GABA_ to more negative values (*p<0.05). n = 5 cells, P0–P3 VGAT-Venus mice. (C) Western blot analysis of NKCC1 and β-actin from the cortex of P0 wild type and VGAT-Venus mouse (n = 3 cortices, respectively). Quantitative analysis of the expression level of NKCC1 were normalized to that of β-actin. (D) Western blot analysis of KCC2 and β-actin from the cortex of P0 and P20 wild type and VGAT-Venus. Quantitative analysis of the expression level of KCC2 were normalized to that of β-actin (n = 3 cortices, respectively). NKCC1 is equally expressed in wild and VGAT-Venus mice, but KCC2 is scarcely detected at P0. Each column indicates mean ± SEM. (E–G) Three horizontal sections containing the MZ (E, F) and the CP (G) from a P0 VGAT-Venus mouse are shown. Sections with Venus fluorescence were counterstained for NKCC1 (E) or KCC2 (F, G). Arrowheads indicate VGAT-Venus positive neurons that were also positive for NKCC (E) or KCC2 (G). Note that NKCC1, but not KCC2, was observed in the MZ (E, F). Scale bar = 30 µm.

To confirm that GABA results in a depolarization of the GABAergic interneurons during multidirectional tangential migration, we made horizontal brain slices containing the MZ, and measured the GABA_A_R reversal potential of VGAT-Venus using gramicidin-perforated patch-clamp recordings which maintain [Cl^−^]_i_ intact [26]. Ramp voltage step were applied to Venus positive cell (Fig. 3A) in the absence and presence of GABA application. Based on the resulting current-voltage relationships, the reversal potential of GABA_A_R response was used to estimate the Cl^−^ equilibrium potential (E_Cl_), which was measured as −43.4±6.1 mV, (Fig. 3B; n = 5 cells). This E_Cl_ of GABAergic interneurons, was more depolarized than the resting membrane potential (V_rest_) measured in the same cells (Fig. 3B, −58.3±4.4 mV, n = 5 cells, p<0.05). Bumetanide shifted E_Cl_ negatively to −60.3±5.9 mV (n = 5 cells, Fig. 3B), closed to V_rest_. This result suggests that NKCC1 activity maintains a high [Cl^−^]_i,_ which is responsible for GABA induced membrane depolarization in immature GABAergic interneurons.

### Expression level of cation-chloride transporters

The [Cl^−^]_i_ has been reported to be predominantly regulated by two cation-chloride cotransporters in immature and mature neurons [27]. NKCC1, as mentioned previously, transport Cl^−^ into neurons while the K^+^-Cl^−^ cotransporter 2 (KCC2) excludes Cl^−^ from neurons [28]. A developmental change in the expression and function of these transporters switches the GABA response from depolarization to hyperpolarization [29]. We therefore examined the protein level of NKCC1 between wild type and VGAT-Venus. NKCC1 was equally expressed in the two strains (Fig. 3C). The expression levels of KCC2 have been reported to be elevated in cortical GABAergic interneurons during early developmental stages, with a critical role in regulating the termination of migration [16]. In the present experiment, however, the expression level of KCC2 at P0 was very low, under detectable levels, while marked expression was observed in more mature neurons from wild type and VGAT-Venus mice under the same conditions (Fig. 3C, 3D). To further determine the extent of NKCC1 and KCC2 expression in individual interneurons in the MZ, we immunostained for these transporters in slices from fixed brains from P0–P3 VGAT-venus mice (Fig. 3E–G). No GABAergic interneurons in the MZ showed any expression of KCC2 whereas a substantial proportion of GABAergic interneurons in the CP showed staining for KCC2 (Fig. 3F, 3G). In contrast, most GABAergic interneurons in the MZ stained positive for NKCC1.

### Different migration rate between VGAT-Venus and GAD67-GFP mice

To investigate the effect of extracellular GABA concentration on multidirectional migration rate, we employed another transgenic strain, the GAD67-GFP knock-in mice (GAD67-GFP mice), which specifically express GFP in GABAergic interneurons. In this strain, there is a decrease of GABA content in the immature cortex, caused by the inactivation of a single GAD67 gene allele [30].

**Figure 4:**
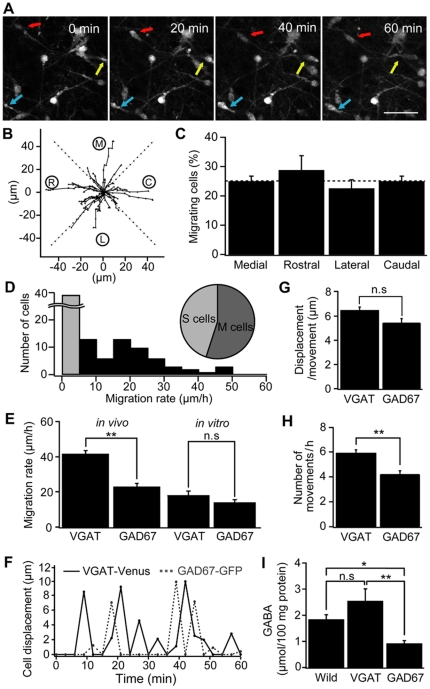
Comparison of multidirectional migration between GAD67-GFP and VGAT-Venus mice. (A) Time-lapse images of migrating neurons in the MZ of a P2 GAD67-GFP mouse. Times shown at the upper right represent the time elapsed after starting the observation. Each GABAergic interneuron is marked by a red arrow. Scale bar = 40 µm. (B) The trajectory of migrating neurons in GAD67-GFP mice (n = 45 cells, 4 animals at P0–P3). The initial location of each neuron at start time of imaging is normalized to the center position. Black filled circles in each line represent the location of each neuron observed at 3 minutes interval. M: medial; C: caudal; L: lateral; R: rostral. (C) The proportion of neurons moved toward each direction. Dotted line is drawn at 25% level. (D) Histogram of the migration rate. Gray bar indicates the number of stationary cells which migrate less than 5 µm during 1 hour. Inset indicates the proportion of cells showing a moving or stationary behavior. (E) Comparison of average displacements per 1 hour between VGAT-Venus and GAD67-GFP mice *in vivo* and *in vitro*, using horizontal slices of neocortex. (F) Cell displacements plotted as a function of time which shows the discontinuous translocation of the cells. Time interval between each observation is 3 minutes. (G) Average distance during each moving phase of VGAT-Venus and GAD67-GFP mice *in vivo* (p>0.05). (H) The frequency of moving phase of VGAT-Venus and GAD67-GFP mice *in vivo* (**p<0.01). (I) Summary graph of GABA contents by using high-performance liquid chromatographic (HPLC) analysis in the cerebral cortex of neonatal (P0–P3) wild type (n = 10), VGAT-Venus (n = 9) and GAD67-GFP (n = 9) mice (*p<0.05, **p<0.01). The levels of GABA were expressed as micromoles/100 mg proteins. Each column indicates mean ± SEM.

We performed an *in vivo* observation of the movements of GABAergic interneurons in GAD67-GFP mice and quantified their migratory behavior as done in VGAT-Venus mice (Fig. 4A). Migration of GABAergic interneurons in the MZ showed a similar lack of preference for direction, as seen in VGAT-Venus mice (Fig. 4B, 4C, medial; 24.9±1.8%, rostral; 28.7±5.0%, lateral; 22.5±3.0%, caudal; 24.9±1.8%). Furthermore, the histogram of migration rate also showed a broad distribution among this population and again nearly half of them did not migrate significantly (Fig. 4D, n = 4 animals, 55.0±7.7% for moving cells and 45.0±8.4% for stationary cells). However, there was a significant difference in the average migration rate between the two strains *in vivo* (Fig. 4E, 41.5±1.8 µm/hr for VGAT-Venus; n = 61 cells, 5 mice and 22.8±2.1 µm/hr for GAD67-GFP; n = 45 cells, 4 mice, p<0.05). In contrast, when we analyzed migration rate *in vitro* using brain slices from each strain, both species showed a reduced migration and there was no significant difference between the two (Fig. 4E, n = 139 cells, 7 cortices for VGAT-Venus, n = 122 cells, 7 cortices for GAD67-GFP). To further characterize the behavior of each cell that undergoes multidirectional migration, we analyzed the movement *in vivo* as a function of time using time-lapse sequences (Fig. 4F). GABAergic interneurons in both GAD67-GFP and VGAT-Venus mice showed discontinuous, saltatory movements. There was no significant difference in the distance of each single movement phase between the two strains (Fig. 4G, 6.47±0.28/hr for VGAT-Venus; n = 61 cells, 5 mice and 5.44±0.36/hr for GAD67-GFP mice; n = 45 cells, 4 mice). However, the frequency of these movement phases was significantly less in GAD67-GFP mice than that in VGAT-Venus mice *in vivo* (Fig. 4H, 5.90±0.27 for VGAT-Venus and 4.20±0.27 for GAD67-GFP mice). This result suggests that the lower level of ambient GABA reported for the GAD67-GFP mice (and also expected in the *in vitro* conditions), decreases migration rate by disturbing the trigger of movement phase.

To confirm this correlation between the GABA content and migration rate, we measured the levels of GABA in the cerebral cortex of both strains using high performance liquid chromatography (HPLC). The forebrain of P0 mice, including the cerebral cortex, contained GABA at a concentration of 2.54±0.47 µmol/100 mg in VGAT-Venus (Fig. 4I, n = 9), 0.91±0.12 µmol/100 mg in GAD67-GFP (n = 9), and 1.83±0.19 µmol/100 mg protein in wild-type mice (n = 10). The GABA content in GAD67-GFP mice was significantly lower than those in wild type and VGAT-Venus mice (p<0.05, one-way ANOVA followed by post-hoc Tukey-Kramer test). In contrast, the GABA content was not different between wild type and VGAT-Venus mice (p>0.05).

**Figure 5:**
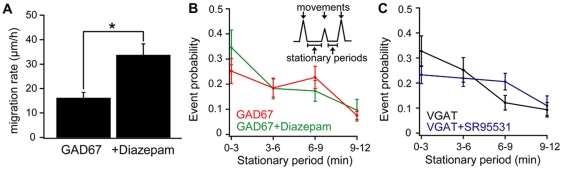
The effect of GABA _A_ receptor modulators on migration properties *in vivo.* (A) *In vivo* application of 2 mM diazepam in GAD67-GFP mice (GAD 67, n = 3 mice) significantly increased the migration rate of GABAergic interneurons. (*p<0.05). (B) Probability plots of the duration of stationary periods for neurons in GAD67 mice (n = 3 mice, red) and with diazepam (GAD67 +diazepam, green, n = 3 mice). Diazepam increased the probability of neurons having a shorter stationary period. (C) Probability plots of the duration of stationary periods for neurons in VGAT-Venus mice (n = 5 mice, black) and with SR95531 (VGAT + SR95531, purple, n = 5 mice). SR95531, a GABA_A_ receptor antagonist increased the probability of long stationary periods. Each point in B and C indicates mean ± SEM of 15–50 events.

These results confirmed the correlation between the GABA content in VGAT-Venus and GAD67-GFP mice *in vivo*, and the migration rate. To further confirm that the activation of GABA_A_R by ambient GABA in GAD67-GFP mice has a critical role in migration rate, we applied diazepam, which increases the activation by GABA of GABA_A_Rs containing α/γ2 subunits [31], [32]. Diazepam increased the migration rate (Fig. 5A) to 33.7±4.6 µm/hr for the diazepam treated group from 16.1±1.8 µm/hr for the DMSO control group, and reduced the duration of the stationary phase between each movement in GAD67-GFP mice (Fig. 5B). Furthermore, we found that the application of the GABA_A_R antagonist, SR95531, also shortens the stationary phase in VGAT-Venus mice (Fig. 5C).

## Discussion

In the present study, we developed *in vivo* imaging for immature mouse cortex with two-photon microscopy and quantified the migratory behavior of GABAergic interneurons in the MZ. To specifically identify the GABAergic interneurons, we employed two strains of mice, VGAT-Venus transgenic and GAD67-GFP knock-in mice in which the fluorescence is specifically expressed in GABAergic interneurons. Furthermore, we utilized the fact that the two strains have significant differences in their GABA content within the cerebral cortex (Fig. 4). Although each individual neuron seemed to move in a specific direction, the population of migrating neurons equally moved in all directions. Nearly half of neurons were stationary during the recording session. These features were similar in both strains. In contrast, the migration rate of VGAT-Venus mice was significant higher than that of GAD67-GFP mice, suggesting that ambient GABA mediates the multidirectional tangential migration. Previous reports have demonstrated an increased expression of specific GABA_A_ receptor subunits (α1, α2, α5, γ2, and γ3) in mouse neocortical interneurons during tangential migration, coupled with altered pharmacological properties [32]. There remains the possibility that different properties of GABA_A_R receptors in the two mice strains may also contribute to the reduced migration rate in the GAD67-GFP mice observed here.

A role for GABA in the migration of cortical GABAergic interneurons has been previously discussed [14]–[17], [33], [34]. In this study, we investigated the role of GABA for multidirectional tangential migration in the MZ by combining *in vivo* and *in vitro* preparations. *In vivo* imaging of genetically defined GABAergic interneurons in the developing cerebral cortex revealed that multidirectional migration rate was enhanced by the membrane depolarization resulting from activation of GABA_A_Rs. GABA-induced depolarization is caused by NKCC1 activity causing a high intracellular Cl^−^ concentration. We also show that intracellular Ca^2+^ was critical for migration, and the GABA_A_R induced depolarization may enhance migration by increasing intracellular Ca^2+^.

### *In vivo* imaging for immature brain

Some previous papers have also taken up the challenge to undertake *in vivo* time-lapse imaging of neuronal migration [7], [35]. Now we have designed an improved experimental configuration to archive stable *in vivo* time-lapse imaging to quantify the role of GABA signaling on multidirectional tangential migration of immature cortical GABAergic interneurons. On average, the *in vivo* population of migrating interneurons has no preference in direction and this was roughly similar to that in previous observations employing *in vitro* preparation [6]. However, the *in vivo* migration rate was much higher than that found *in vitro* (Fig. 4E), suggesting that some intrinsic factor(s) that regulate migration are disturbed in the slice preparation.

### Multidirectional tangential migration in the postnatal cortex

Whereas many studies have examined interneuronal migration in the embryonic cortex [1], [2], [8], few studies have reported on migration in the postnatal mouse cortex. Indeed, tangentially migrating interneurons are depleted from the IZ by P0.5 [12] while the density of interneurons in the MZ decreases between P0.5 and P7.5 in a non apoptotic manner [13], which implies that the MZ is a dominant cortical layer for GABAergic interneurons migration during early postnatal development. Consistent with the previous report [13], we observed that the cell density in the MZ imaging plane decreased between P0 and P3 in both control and ACSF treated VGAT-Venus mice groups. Despite a reduction of the total number of cells, the proportion of stationary cells was preserved, indicated that the most stationary cells observed at P0 in the MZ eventually migrated towards the deep cortical plate and their final positions. These results suggest two distinct hypotheses. Firstly, there is a waiting period for entering the CP. Several types of developing axons are also known to stop growing and wait for the maturation of their target [36], [37]. By analogy, GABAergic interneurons in the MZ may require further maturation before continuing their migration. Secondly, the migrating GABAergic interneurons in the MZ may release signals which suppress the migration of neighboring cells. As a result, the proportion of activating (moving) cells in the MZ is maintained as constant.

### The contribution of GABA for intracellular Ca^2+^ and multidirectional migration

It is well established that activation of GABA_A_R has a depolarizing effect in immature cortical neurons [38]. In rodents, this depolarizing action of GABA changes at the 2nd postnatal week into a hyperpolarization [39] due to a developmental upregulation of the neuronal chloride-extruding molecule, KCC2 [28] and a downregulation of inward Cl^−^ transporter, NKCC1 [40]. Although the exact downstream mechanisms have not been fully elucidated, previous observations [41] suggests that GABA_A_R activation profoundly regulates the migration process by altering [Ca^2+^]_i_ levels. Similarly, GABAergic interneurons in the MZ during multidirectional migration *in vivo* responded to the specific GABA_A_R antagonist, SR95531, and to the intracellular Ca^2+^ chelator BAPTA-AM with a reduction in migration rate (Fig. 2). This suggests that the activation of GABA_A_R in immature neurons produces a depolaration-induced increment of [Ca^2+^]_i_, secondary to the more depolarized Cl^−^ equilibrium potential at this developmental stage [39]. A release from intracellular Ca^2+^ store, e.g. Ca^2+^ induced Ca^2+^ release, has also been reported as crucial for migration in cerebellar granule cells [42].

### Multidirectional dispersion enhanced by ambient GABA

Previous studies reported that GABAergic interneurons wait in the MZ for 24–48 hours before entering the cortical plate [10], [43]. These reports suggested that multidirectional tangential migration in the MZ contributes to the ultimate dispersion of GABAergic interneurons throughout the cortical area. In the present study, we proposed that, in living mice, ambient GABA accelerates the rate of multidirectional tangential migration caused by its depolarizing effect. Indeed, several studies have reported that the activation of GABA_A_R regulates neuronal proliferation [44], migration [16], [41] and differentiation [45] in the developing cerebral cortex. Thus, the function of GABA signaling during development serves to balance the spatial arrangement of glutamatergic and GABAergic neurons which compose mature cortical neural circuits. In addition, the *in vivo* imaging techniques for immature brain used in this study will strongly contribute to further research into the development of neuronal circuits.

## Materials and Methods

The experimental protocols in this study were approved by the Animal Research Committee of the National Institutes of Natural Sciences (approval number 10A179). All efforts were made to minimize the suffering and number of animals used in this study.

### Animals

Experimental animals were obtained by mating female C57BL/6 mice with hemizygous male VGAT-Venus transgenic mice [23] or with heterozygous male GAD67-GFP knock-in mice [30]. The experiments were performed using mice species and ages as follows: 1) two-photon imaging, P0–P3 VGAT-Venus + GAD67-GFP mice; 2) high performance liquid chromatography (HPLC), P0 VGAT-Venus + wild type mice; 3) electrophysiology, P0–P3 VGAT-Venus mice; 4) western blotting, P0 and P20 VGAT-Venus + wild type mice; 5) immunohistochemistry, P0 VGAT-Venus mice.

### Animal surgery for *in vivo* imaging

Animals were anesthetized by i.p. injection of ketamine/xylazine (130/19.5 mg/kg of body weight). Depth of anesthesia was assessed by monitoring the tail-pinch reflex and respiration rate. After removing the skin from the temporal and parietal sides of the head, the mice were transferred into the set-up and placed onto a warming plate (37°C). The stainless steel restraint bars were then fixed on the skull with dental cement and cyanoacrylate adhesive (Vetbond; 3M, St. Paul, MN, USA). A custom-made recording chamber with a hole in the center was placed on the restraint bars, so as to not exert any pressure on to the skull and brain. The gap between the recording chamber and the skull was filled with low melting-point agarose gel (SeaPlaque, Lonza Rockland, ME, USA) made at 2% w/v with saline. The recording chamber was perfused with a warm (35°C) distilled water to help maintain the body temperature at the imaging area.

### *In vivo* two-photon imaging

Animals were anesthetized either by inhalation of isoflurane (1.0% in pure O_2_) or i.p. injection of ketamine/xylazine during imaging. Imaging was performed by using a custom built two-photon laser-scanning microscope based on a mode-locked laser system operating at 950 nm, 80-MHz pulse repetition rate, 100-fs pulse width (Mai Tai HP, Spectra Physics, Mountain View, CA, USA) and a laser scanning system (Olympus FLUOVIEW, Olympus, Tokyo, Japan) coupled to an upright microscope (BX61WI, Olympus) and equipped with water-immersion objectives: ×25, 1.05 numerical aperture (Olympus). The coordinate of the imaging area was as follows: 4.5 mm posterior to the rostral end of the brain, 1.0 mm lateral to midline. This region develops into the mediomedial secondary visual cortex (V2MM) in adult mouse, which was identified based on the comprehensive atlas of developing mouse brain [46]. Images were taken from the MZ (at a depth of 0–50 µm under the skull) at 3 min intervals. A stack of images for tangential migration was the sum of 10×0.5 µm sections (total thickness 5 µm), advancing in the Z axis. The average power delivered to the brain was <30 mW. Images were analyzed off-line with MetaMorph software (Molecular Devices, Downingtown, PA, USA).

### Measurement of GABA content

To measure GABA levels in the cerebral cortex of immature mice, high performance liquid chromatography (HPLC) was employed. Cortical tissues from P0 mice were dissected and homogenized with 0.1 M perchloric acid to destroy the three dimensional structures of proteins and make amino acids soluble. The homogenates were centrifuged at 10000 g for 15 min at 4°C and then the supernatant was neutralized with 0.1 M sodium carbonate. After addition of sodium bicarbonate, the samples were filtered by using Ultrafree-MC filters (Millipore Corp., Bedford, MA, USA). Amino acids in the samples were derivatized to o-phthalaldehyde. An octadecylsilyl column was used and kept at 30°C in a column oven (ATC-300; Eicom, Kyoto, Japan). The mobile phase consisted of (in mM) 50 NaH_2_PO_4_ 2H_2_O at pH3.5, 0.015 EDTA 2Na, and 50% methanol. A flow rate of 0.5 ml/min was maintained using an isocratic pump (EP-300, Eicom). Protein concentrations were measured by a Quick Start protein assay kit (Bio-Rad Laboratories, Hercules, CA, USA) which employed bicinchoninic acid protein assay reagent with bovine serum albumin as a standard.

### Electrophysiology

Animals were anaesthetized with 130/19.5 mg/kg ketamine/xylazine (i.p.) and sacrificed by decapitation, and brains were rapidly removed and transferred into ice-cold ACSF (124 mM NaCl, 2.5 mM KCl, 1.2 mM KH_2_PO_4_, 1 mM MgSO_4_, 2 mM CaCl_2_, 10 mM glucose, 24 mM NaHCO_3_). This solution was adjusted to pH 7.4 with tris(hydroxymethyl)aminomethane (Tris-base). Horizontal slices were cut by a vibratome (VT1000S; Leica, Nussloch, Germany) in ice-cold ACSF bubbled with 95% O_2_ and 5% CO_2_. The neocortical 300 µm-thick slices were kept in control incubation solution (124 mM NaCl, 2.5 KCl, 1.2 mM NaH_2_PO_4_, 24 mM NaHCO_3_, 2.4 mM CaCl_2_, 1.3 mM MgCl_2_ and 10 mM glucose) saturated with 95% O_2_ and 5% CO_2_ at room temperature (21–24°C) for at least 1 hour before being transferred into the recording chamber. Gramicidin-perforated patch-clamp recordings were done on Venus-positive neurons in the MZ at room temperature in an incubation solution bubbled with 95% O_2_/5% CO_2_ (solution flow rate 0.8 ml/min) and with slices anchored with silver bars. Patch pipettes were made from borosilicate capillary glass in two stages on a vertical pipette puller (PC-10, Narishige, Tokyo, Japan). The resistances between the recording pipettes filled with internal solution and the reference electrode in the bubbled external ACSF solution were 4–6 MΩ. Neurons were visualized under phase contrast on an in upright microscope (ECLIPSE E600FN, Nikon, Japan). Current and voltage were continuously monitored on a pen recorder (WR3320, Graphtec, Tokyo, Japan) and stored on a computer equipped with pCLAMP8.2 software (Axon Instruments). Venus or GFP positive cells were excited by a mercury lamp (HBO MERCURY SHORT ARC, OSRAM, Germany) and recordings were only done on these fluorescently labeled cells. GABA-activated currents were measured with a patch-clamp amplifier (Multi Clamp 700B, Axon CNS, Molecular Devices), and recorded with a sampling frequency of 10 kHz after low-pass filtering at 2 kHz. The neurons were voltage-clamped at a holding potentialof −60 mV. E_GABA_ was measured by using voltage ramps (from −80 to −20 mV, 3 s duration), from a holding potential (V_H_) of −60 mV, applied before and during 10 µM GABA application. GABA was puffed onto the soma using a microinjector (IM 300, Narishige, Tokyo, Japan). The internal (patch pipette) solution for perforated patch clamp recordings contained 60 mM potassium methanesulfonate, 90 mM KCl, 10 mM HEPES (pH 7.2). Gramicidin was dissolved in dimethyl sulfoxide (DMSO) to prepare a stock solution of 10 mg/ml, and then diluted to a final concentration of 30 µg/ml in the pipette solution. The gramicidin solution was prepared just before use. The series resistance typically reached a steady level below 30 MΩ within 40 mins after making the GΩ seal. During recordings, the series resistance errors were electronically compensated for by 70–80%. To minimize series resistance errors, current responses were kept small by restricting the voltage range and using a submaximal GABA concentration. With a peak current of 100 pA, the uncompensated voltage drop across a 30 MΩ series resistance would be less than 1 mV.

### Western blotting

Proteins were separated using 7.5% acrylamide gels by SDS-PAGE. The gels were transferred to an Immobilon-P (Millipore). The blots were blocked in 1% bovine serum albumin and incubated overnight with primary antibody at 4°C. They were then incubated with horseradish peroxidase-conjugated secondary antibody (GE Healthcare UK Ltd., Buckinghamshire, England) for 1 hour at room temperature. Enhanced chemiluminescence (ECL, GE Healthcare) exposure on instant film and an ECL mini-camera luminometer were used to visualize labeled protein. The following primary antibodies (and their dilutions) were used: anti-NKCC1 (1∶200, Millipore), anti-KCC2 (1∶1000, Millipore). The optical densities of each band were analyzed with Scion Image software with a gel-plotting macro program. The total NKCC1 and KCC2 band densities were normalized to the β-actin band density. Western blottings were repeated at least three times to ensure the reproducibility of results.

### Immunohistochemistry

P0 VGAT-venus mice were anaesthetized with 130/19.5 mg/kg ketamine/xylazine (i.p.) and sacrificed by decapitation. Brains were immediately excised and fixed overnight in 4% paraformaldehyde in 0.1 M phosphate buffer (PB) at 4°C. Thereafter, 20 µm horizontal slices of the neocortex were cut using a vibratome (VT1000S; Leica, Nussloch, Germany). After washing in PB, slices were incubated in blocking solution (10% normal goat serum, 2% bovine serum albumin, 0.5% Triton X-100 in 0.1 M PB) containing primary antibodies overnight at 4°C. NKCC1 was stained with a monoclonal mouse antibody (T4, Developmental Studies Hybridoma Bank, Iowa City, IA, 1∶800 dilution) for 24 h at 4°C. KCC2 immunostaining used a polyclonal rabbit antibody (Upstate, Temecula, CA, 1∶1000 dilution) for 24 h at 4°C. Alexa633-bound goat anti-mouse and Alexa594-bound goat anti-rabbit fluorescent secondary antibodies (1∶200 dilution; Invitrogen, San Diego, CA) were then applied for 3 hrs at room temperature. Fluorescent images were acquired with a confocal laser scanning microscope (Leica TCS SP2, Wetzlar, Germany).

### Data analysis and statistics

All values are given as means ± SEMs. A two-tailed Student's t test was used to determine significant differences between different conditions. For multiple groups, one-way ANOVA with post-hoc Tukey-Kramer test was used. The level of significance was set at p<0.05 for all tests.

### Drugs

Drugs used in the present study were SR95531, BAPTA-AM, gramicidin D, bumetanide, diazepam and GABA from Sigma (St. Louis, MO, USA) and sulforhodamine 101 from Invitrogen (Eugene, OR, USA). Final drug concentrations were made up from stock solutions just before use.
